# Cranioplasty for a Growing Fracture of the Skull: A Case Report

**DOI:** 10.7759/cureus.30271

**Published:** 2022-10-13

**Authors:** Vaidehi Mendpara, Sweta Sahu, Krupaa Madhu, Sumaiya Tarannum Shaik, Manasvi Reddy Maram, Balaganesh Natarajan, Swetha Movva, Anam Sayed Mushir Ali, Dharmesh R Chauhan

**Affiliations:** 1 Medicine and Surgery, Government Medical College, Surat, IND; 2 Surgery, Jagadguru Jayadeva Murugarajendra (JJM) Medical College, Davanagere, IND; 3 General Surgery, Gujarat Medical Education & Research Society (GMERS) Medical College, Gandhinagar, IND; 4 Medicine, Osmania Medical College, Hyderabad, IND; 5 General Surgery, Osmania Medical College, Hyderabad, IND; 6 Neurology, St. George's University School of Medicine, St. George's, GRD; 7 Pediatrics, Narayana Medical College, Nellore, IND; 8 Medicine, Indian Institute of Medical Science and Research, Aurangabad, IND; 9 General Surgery, Government Medical College, Surat, IND

**Keywords:** duraplasty, pediatric neurosurgery, growing skull fracture (gsf), posterior fossa decompression and duraplasty, titanium cranioplasty

## Abstract

Pediatric growing skull fractures are complications that usually occur due to delays in management. In this report, we present the case of a three-year-old girl who was brought to the outpatient department with a complaint of swelling in her scalp. The patient had a history of swelling after suffering a head injury at the age of six months. There was no history of specific neurological impairments or seizures, despite the swelling being reported to have grown gradually in size. The current case is being reported since early evaluation of pediatric patients with a head injury, regardless of any neurological shortfalls, should be thoroughly worked up to prevent any progressively growing cranial defects. The subtlety of these pediatric head injury cases tends to cause misdiagnosis, which can delay management and can cause complications, as with this patient. Extended observation, intensive supportive care, and neurosurgery are considered when dealing with these seemingly innocuous cases.

## Introduction

Blunt fractures to the calvarium are the main cause of trauma-related mortality in children. The most commonly affected bone is the parietal, followed by the occipital, frontal, and temporal bones. Almost 85% of pediatric skull fractures are linear [1]. Rarely, growing skull fractures (GSFs) are a complication in children who sustain head trauma, which can later manifest as neurological problems. Neurological symptoms could include cranial nerve abnormalities such as hearing defects, anosmia, visual defects, paralysis, or even facial numbness. Other symptoms may also include seizures, vomiting, and discharge from the ear or nose. Reports put the incidence of expanding skull fractures in childhood between 0.05% and 0.1% [2]. The pathophysiology of GSFs begins with a skull fracture that is accompanied by a tear in the dura mater. The pulsatile nature of the brain, along with herniated elements, inhibits osteoblast healing properties, which further enables the fracture in the skull to worsen and grow [3]. Herniated tissue exerts constant pressure, which also advances the fracture line. If incorrectly evaluated, even the most minor blunt trauma can induce growing skull fractures or craniocerebral erosions. Craniocerebral erosions are a complication that is sometimes termed a leptomeningeal cyst, as they are often associated with cystic masses filled with cerebrospinal fluid (CSF) [4].

## Case presentation

**Figure 1:**
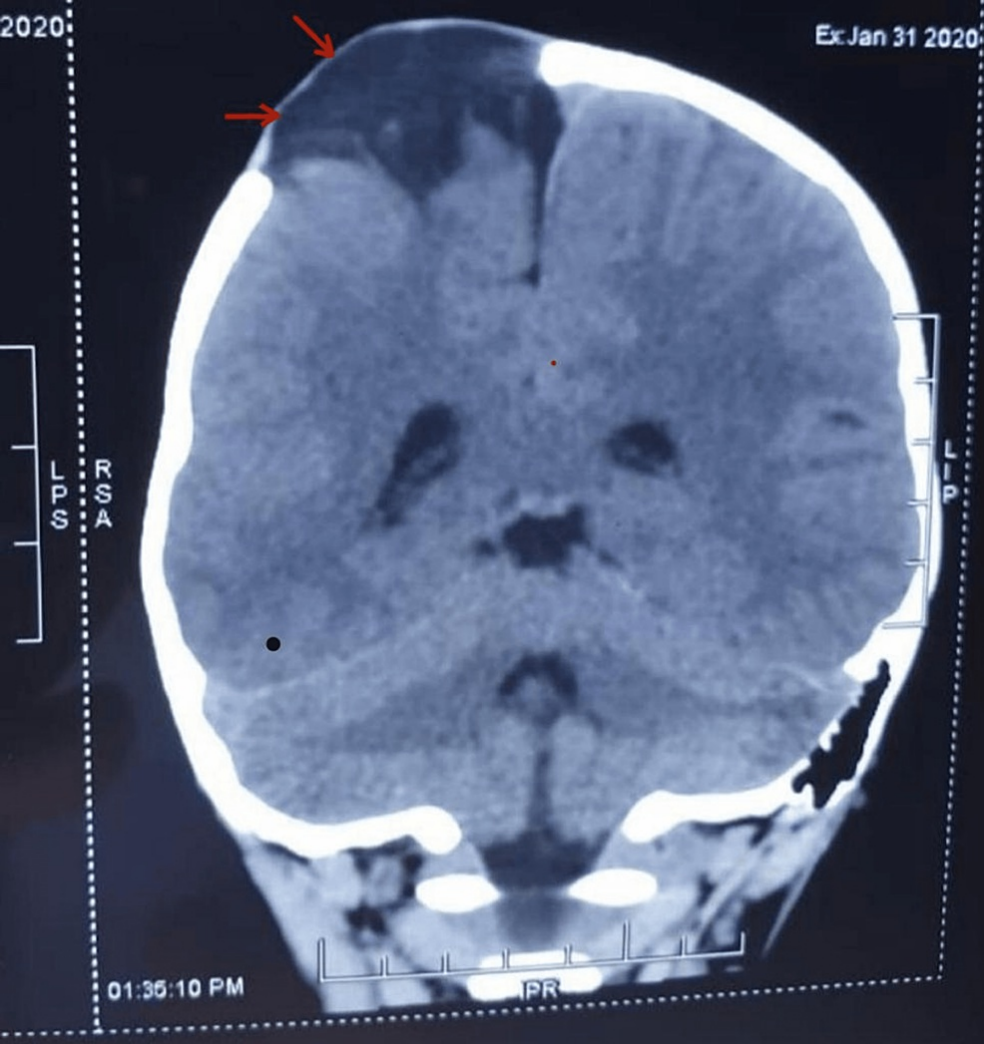
A plain computed tomography scan of the brain showing the defect in the temporoparietal region of the brain

A three-year-old female reported to the outpatient department (OPD) with a two-and-a-half-year history of progressively growing scalp swelling. There had been no prior history of seizures or localised neurological impairment. The patient developed right parieto-temporal hypertrophy ever since sustaining head trauma at the age of six months. At the time of the clinical assessment, the patient presented with a deformed skull, a massive pulsatile and pain-free swelling lesion in the right parietal region, and left-sided hemiparesis. Computed tomography (CT) revealed a defect in the right temporoparietal area, through which a cystic herniation had occurred (Figure 1).

**Figure 2:**
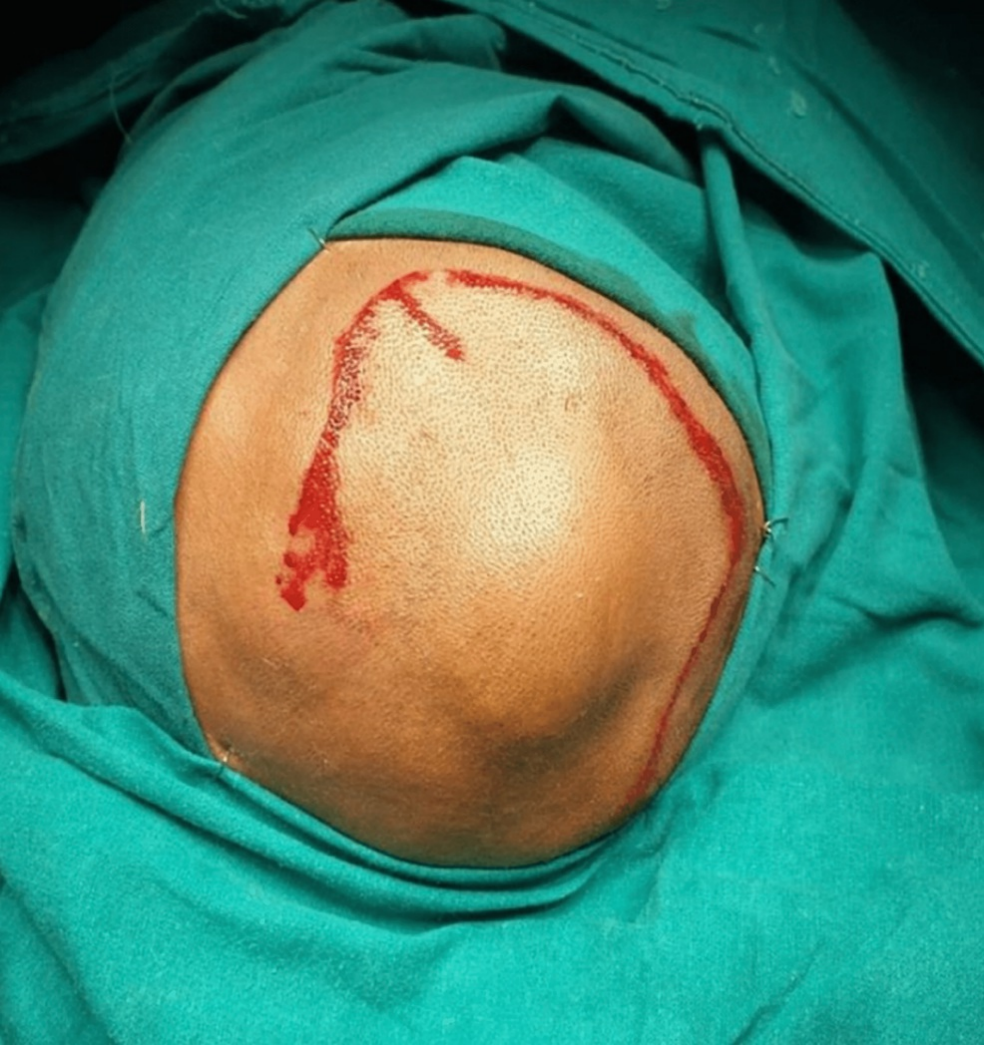
The swelling, and operative incision for the surgery

**Figure 3:**
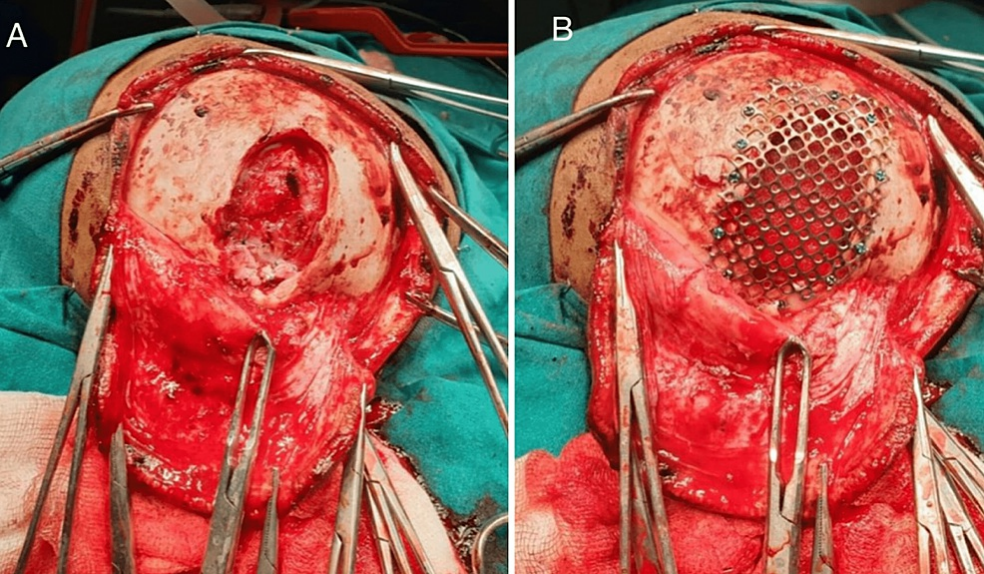
Intra-operative images showing the (A) bone defect along with the lacerated dura mater and (B) procedure of cranioplasty using the titanium mesh for closing the bone defect

Routine laboratory tests yielded normal results. It was determined that surgical repair was necessary (Figure 2). The surgical procedure involved reconstructing the dura and employing the titanium mesh to fix the bone defect (Figure 3).

The child was discharged hours after surgery without complications. After four months of follow-up, the patient had an excellent cosmetic result and a satisfactory functional result.

## Discussion

Growing skull fractures, though uncommon, are still a significant clinical consequence of trauma-related skull fractures in children under three. A fall-related skull fracture is the most frequent factor in numerous recorded case series [5]. The parietal bone is the most frequently damaged region [6]. The exact mechanisms behind the development of increasing skull fractures are not well understood. An intracranial hypertension syndrome and CSF pulsation have both been documented, which prevent the fracture margins from healing following a head injury that results in a fracture of the skull bone with an underlying dural rupture. The invagination and entrapment of arachnoids within a diastatic fracture can furthermore result in bone diastasis coupled with leptomeningeal herniation. This can be a life-threatening condition [7,8]. It is essential to diagnose a GSF as early as possible. Radiological diagnostic methods are helpful. X-ray imaging can reveal fracture lines and abnormalities in the bone. In our case, CT of the skull helped in confirming the diagnosis. CT scans show the precise position and the size of a bone defect and the possibility of brain tissue injury [9,10].

A study on seven patients, based on CT, surgical findings, etiopathogenesis, and necessary therapeutic approaches, distinguished between three primary forms of GSFs: leptomeningeal cysts, in three patients; damaged and gliotic brain, in three patients; and porencephalic cysts extending through the skull defect into the subgaleal region, in two patients [11]. The first surgical step in treating this issue is a duraplasty, as we did in our case. First, we should examine the defect before performing duraplasty. In the case of a herniated gliotic parenchymal or leptomeningeal cyst, however, the surgical approach may involve the excision of both the tissue and the cyst. Cranioplasty should be done after duraplasty [11]. The mortality rate (due to anaesthesia and meningitis) ranges from 0% to 8%. Surgery should be performed right away to safeguard the brain and limit seizure frequency [9]. Pericranial autografts were chosen above the other possibilities during the duraplasty treatment because they not only provide a watertight dural closure but also lower the risk of adhesions, infections, and rejection. Fascia lata, bovine pericardium, fetal bovine tissue, processed collagen matrix, and synthetic materials are a few more grafts that may be employed. Because of its excellent biocompatibility, low infection rate, outstanding mechanical strength, and affordable price, titanium mesh was chosen as the best implant material for cranioplasty. Dural and cranial abnormalities that reach the venous sinuses should not be operated on since they are life-threatening and will not change if left untreated. Any surgical wound dripping CSF in the recovery area is not a cause for alarm. However, our patient did not experience any side effects, and recovery went exceptionally smoothly.

## Conclusions

Growing skull fractures can result in severe neurologic problems; however, our patient did not have any such condition. Imaging methods, such as CT and MRI, are good at catching GSFs early on. In our case, the outcome was favorable because of the correct line of diagnostic approach and treatment. It is therefore advised to screen children at risk of developing the condition by teaching parents about the possible outcome and then closely monitoring those children using clinical and imaging screening. Infant and childhood head trauma should not be taken casually by the parents as the patients from the affected age group are themselves unaware of the consequences.
